# Typical Radiographic Findings of Dentin Dysplasia Type 1b with Dental Fluorosis

**DOI:** 10.1155/2013/902861

**Published:** 2013-12-03

**Authors:** S. Venkata Suman, Raviraj Jayam, B. Vijay Kumar, Suresh Dirasantchu, K. Venkata Suneel Kumar, Sameeulla Sk

**Affiliations:** ^1^Department of Oral Medicine and Radiology, CKS Theja Dental College, Hospital and Research Centre, Tirupati, Andhra Pradesh 517501, India; ^2^Department of Oral Medicine and Radiology, Narayana Dental College and Hospital, Nellore, Andhra Pradesh 524001, India

## Abstract

Dentin dysplasia is a rare inherited autosomal dominant disorder characterized by rootless teeth. We hereby report a case of dentin dysplasia type 1b with typical radiographic findings: short and blunt roots, pulpal obliteration, horizontal/crescent shaped radiolucencies in pulp chambers, and multiple periapical radiolucencies. However, the present case did not show the autosomal dominant pattern of inheritance and the patient also exhibited concurrent dental fluorosis, transposition of 13 and 14, and multiple cusps in maxillary first molars. Moreover, on careful review of previously documented cases of radiographs of dentin dysplasia, the horizontal/crescent shaped radiolucencies in pulp chambers are a rare finding, which is characteristically seen in the present case.

## 1. Introduction

Dentin dysplasia (DD) is an autosomal dominant hereditary disturbance in dentin formation, which may present with either mobile teeth or pain associated with spontaneous dental abscesses or cysts. It is a rare anomaly of unknown etiology that affects approximately one patient in every 100,000 [1]. The condition was first described by Ballschmiede, but it was Rushton who termed the condition dentinal dysplasia. This condition is rarely encountered in dental practice. In 1972, Witkop Jr. [2] classified DD into two types: radicular DD as type I and coronal DD as type II. In type I, both the deciduous and permanent dentitions are affected. The crowns of the teeth appear clinically normal in morphology, but defects in dentin formation and pulp obliteration are present. Radiographic examination is important for the identification of DD type I. In the coronal type, the pulps are enlarged and are described as having a “thistle tube” appearance, in permanent dentition. In the deciduous dentition, coronal dentin dysplasia bears a resemblance to dentinogenesis imperfecta type II.

The present case is dentin dysplasia type 1b with typical radiographic findings: short and blunt roots, pulpal obliteration, horizontal/crescent shaped radiolucencies in pulp chambers, and multiple periapical radiolucencies.

Though dentin dysplasia is transmitted as autosomal dominant disorder, the present case did not show the autosomal dominant pattern of inheritance and the patient also exhibited concurrent dental fluorosis, transposition of 13 and 14, and multiple cusps in maxillary first molars. Moreover, on careful review of previously documented cases of radiographs of dentin dysplasia, the horizontal/crescent shaped radiolucencies in pulp chambers are a rare finding, which is characteristically seen in the present case.

## 2. Case Report

A 16-year-old male patient presented with a chief complaint of partial edentulousness due to early shedding of teeth. Patient did not give any history of trauma. Extraoral examination did not reveal any abnormality. On intraoral examination, teeth present were

 17 16 15 13 14 22 23 24 25 26 27
 47 46 45 43 33 34 35 36 37

**Figure 1:**
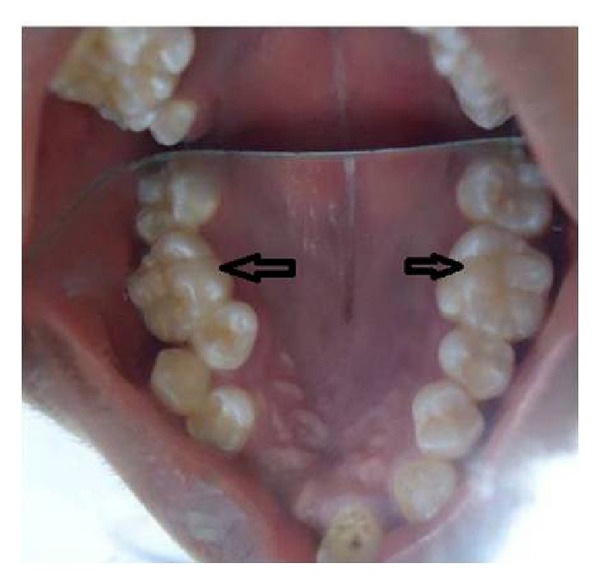
Intraoral photograph showing transposition between 13 and 14 and multiple cusps in occlusal surface of the 1st maxillary molars (black arrows).

**Figure 2:**
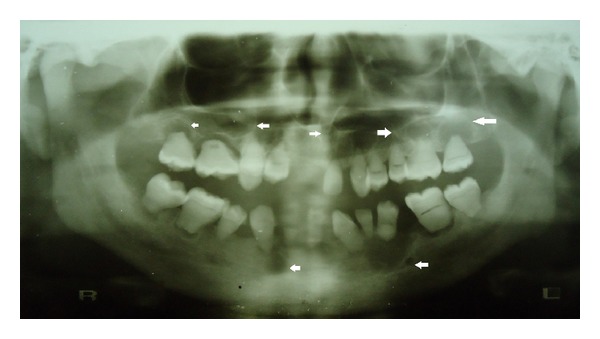
Cropped panoramic radiograph showing generalized short and tapered roots, pulpal obliteration, horizontal/crescent shaped pulpal remnants in pulp chambers, and multiple periapical radiolucencies (white arrows).

**Figure 3:**
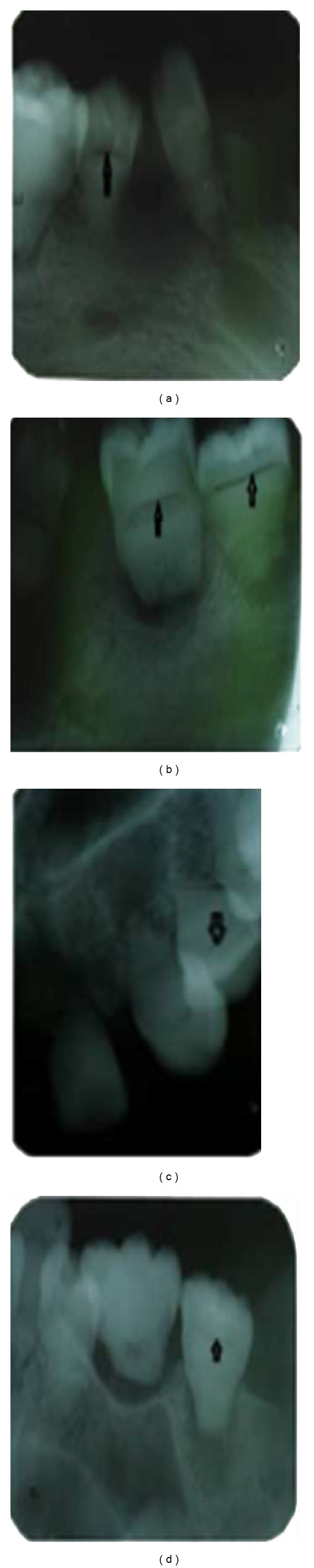
Intraoral periapical radiographs showing horizontal/crescent shaped pulpal remnants in pulp chambers (black arrows).

The majority of the teeth exhibited grade I mobility except for 35 which had grade II mobility. The crowns of teeth showed brownish yellow discoloration with white chalky areas, corresponding to dental fluorosis. The maxillary first molars showed multiple cusps and there was transposition of 13 and 14 (Figure 1). Patient was subjected to panoramic radiograph and full mouth intraoral periapical radiographs. The radiographs revealed features like generalized short and blunt roots, pulpal obliteration, horizontal/crescent shaped pulpal remnants in pulp chambers, and multiple periapical radiolucencies (Figures 2 and 3(a)–3(d)). With the above radiographic findings, this case was diagnosed as dentin dysplasia type 1b. Patient was treated conservatively by advising dietary and oral hygiene instructions, oral prophylaxis, and removable partial denture fabrication for esthetic purpose.

## 3. Discussion

Dentin dysplasia is a rare disorder which is thought to arise due to abnormal differentiation or function of the ectomesenchymally derived odontoblasts [1]. Witkop attributed the ectopic dentin formation to the displacement of internal cells of the developing dental organ and proliferation in the dentin papilla [2]. Logan et al. proposed that obliteration of pulpal space could be due to abnormal degeneration and calcification in dental papilla [3].

Dentin dysplasia is inherited as an autosomal dominant trait. However, in this case none of the family members exhibited radiographic features of dentin dysplasia. This indicated that the patient is a first generation sufferer. Such similar incidence was also reported by Toomarian et al. [4].

Carroll et al. [5] subclassified dentin dysplasia into four types. In type 1a, there are no pulp chambers and root formation, and there are frequent periapical radiolucencies; type 1b has a single small horizontally oriented and crescent shaped pulp, and roots are only a few millimeters in length and there are frequent periapical radiolucencies; in type 1c, there are two horizontal or vertical and crescent shaped pulpal remnants surrounded by a central island of dentin and with significant but shortened root length and variable periapical radiolucencies; in type 1d, there are visible pulp chambers and canal with near normal root length and large pulp stones that are located in the coronal portion of the canal and create a localized bulging in the canal, as well as root constriction of the pulp canal apical to the stone and few periapical radiolucencies.

The present case exhibited classical radiographic features of type 1b. Though the previous literature has documented considerable number of cases of dentin dysplasia type 1, very few of them had successfully demonstrated the horizontal/crescent shaped pulp in their radiographs. Our case clearly demonstrates this feature in addition to other radiographic findings like short roots, pulpal obliterations, and multiple periapical radiolucencies.

The present case also showed other associated clinical findings like dental fluorosis, transposition of 13 and 14, and multiple cusps in maxillary first molars. Dental fluorosis in the present case is attributed to the fact that the patient belonged to the geographical area which is endemic to fluorosis.

Management of dentin dysplasia has always been a challenging task to the dentists. Followup and routine conservative treatment are often advised. Maintenance of periodontal health through adherence to oral hygiene instructions is vital to the survival of mobile teeth. Endodontic treatment is contraindicated in teeth with total obliteration of root canals and pulp chambers [6].

In conclusion, dentin dysplasia is a rare genetic disorder. The present case exhibited characteristic radiographic findings along with other associated clinical findings like dental fluorosis, transposition of 13 and 14, and multiple cusps in maxillary first molars. Also this case did not show the expected pattern of inheritance. Cases of dentin dysplasia have to be treated conservatively to prevent premature exfoliation of teeth.
